# Comparative Studies on Different Extraction Methods of *Centella asiatica* and Extracts Bioactive Compounds Effects on Antimicrobial Activities

**DOI:** 10.3390/antibiotics10040457

**Published:** 2021-04-17

**Authors:** Farhana Nazira Idris, Masrina Mohd Nadzir

**Affiliations:** School of Chemical Engineering, Engineering Campus, Universiti Sains Malaysia, Nibong Tebal, Pulau Pinang, Seberang Perai Selatan 14300, Malaysia; farhana_nazira@yahoo.com

**Keywords:** antimicrobial, bioactive compounds, *Centella asiatica*, extraction, in vitro, in vivo

## Abstract

The interest of consumers in using products containing phytochemicals derived from plants is growing day by day due to the shift of consumers’ preferences from convenience to environmental sustainability. One plant utilized in many products is *Centella asiatica*, a herb commonly used in folk medicine, health supplements, and beauty products. Extraction of bioactive compounds from *C. asiatica* was performed using conventional methods and modern methods (e.g., microwave or ultrasound-assisted and subcritical water extraction). This review summarizes the variety of methods used to extract active compounds from *C. asiatica*, their influence on the bioactive compounds and antimicrobial activity in vitro and in vivo, and the safety and toxicology of *C. asiatica* extract.

## 1. Introduction

Medicinal plants have been used widely in nutraceuticals and cosmeceuticals in which phytochemicals from plants represent natural sources of compounds with several biological benefits. Extracts from plants have been used as additives in different drug formulations to enhance their use as basic health care by 80% of the world’s population [1]. Nowadays, more and more developed countries have started to grow more herbs to turn into plant-based products, therefore, these activities are no longer associated with third-world countries.

*Centella asiatica* is one of the most popular herbs that can be found abundantly in China, Japan, Italy, Sri Lanka, Iran, India, Madagascar, America, Australia, South Africa, Indonesia, and Malaysia [2,3,4,5]. It is a perennial herb in the Umbelliferae family and presents medicinal properties such as anti-inflammatory [6,7,8], anti-ulcer [9], antimicrobial [10,11,12], and memory-enhancing properties [13,14]. It is also used for healing skin diseases such as leprosy, psoriasis, eczema, and itching [9,15,16]. This plant is known as pegaga in Malaysia, codagem in Brazil, tsubo-kusa in Japan, and mandukaparni in India. Other common names are pennywort, gotu kola, and brahmi [5,17].

Different studies published worldwide have reported that up to seventy compounds have been extracted from *C. asiatica* [5]. The most abundant bioactive compounds found in *C. asiatica* are represented by asiaticoside, madecassoside, asiatic, and madecassic acid from the triterpene class [5,6,18,19]. Triterpenes are used in cosmeceuticals mainly for wound-healing, anti-wrinkle, and anti-cellulite effects since they increase the synthesis of collagen and improve the production of fibronectin in human skin fibroblasts [6,20]. Specifically, asiaticoside increases the hydroxyproline content and tensile strength in wound tissue, raises the collagen Type I and III content, expedites the wound-healing process, and induces glycosaminoglycan synthesis [6,20,21,22]. Madecassoside can also stimulate the production of collagen Type III [6]. Other compounds from the saponins group that can be found in a smaller amount in *C. asiatica* are brahmoside, centelloside, glycosides, and alkaloids [5,15]. Flavonoids such as kaempferol, quercetin, rutin, catechin, naringin, and apigenin that contribute to the total phenolic content can also be found in *C. asiatica* [14].

The market for *C. asiatica* is primarily in the Asia Pacific, but keeps rising in the U.S. and Europe due to its wide range of medicinal purposes [23]. Many researchers have studied this plant due to its potential benefits like antioxidant, immune system enhancers, and antimicrobial effects, which could be useful traits when applied in cosmeceutical, pharmaceutical, medicinal, and health-related products. Specifically, *C. asiatica* acts as a natural source of antimicrobial agents, providing an alternative solution to overcome antibiotic resistance, which has become a concern due to the widespread usage of antibiotics [12]. The interest in *C. asiatica* has resulted in patents for topical skincare formulations [24], an oral rinse [25], a lower limb ulcer treatment [26], a memory enhancer in Alzheimer’s disease [27], and a topical hair composition [28]. More than 100 health care formulations based on *C. asiatica* are sold on the market [29], and an asiaticoside and madecassoside content of at least 2% is required for the product to be used in herbal industries. In India, the price for 500–1000 MT of *C. asiatica* is 0.4–0.5 U.S. dollars/kg [30]. The demand for *C. asiatica* in Indonesia has reached 25 tons/year, but the supply is only four tons/year [31]. Since 90% of the supply of *C. asiatica* is exploited from wild plants [32], it is crucial to ensure that harvesting is sustainable to meet the demand. Extraction is also essential to obtain the maximum amount of the desired compounds using the minimum amount of *C. asiatica*.

The typical stages of separating bioactive compounds from plant materials are sample preparation, extraction, and purification. It is crucial to select the appropriate extraction method since more than 60% of the total time is used for the sample preparation stage [33]. Moreover, the right choice of extraction method can improve the extract’s quality and prevent loss of the target compounds. Since extraction is the crucial stage in obtaining the desired compounds from *C. asiatica*, here we review the methods used for the extraction of *C. asiatica*. This review covers the utilization of both simple technologies and advanced extraction techniques that have been reported to get the best yield from the herb, and the in vitro and in vivo antimicrobial activity of the extracts from the selected extraction methods. Finally, the safety and toxicology of the *C. asiatica* extract are further discussed for a better understanding.

## 2. Techniques for Extracting Bioactive Compounds from *Centella asiatica*

**Figure 1:**
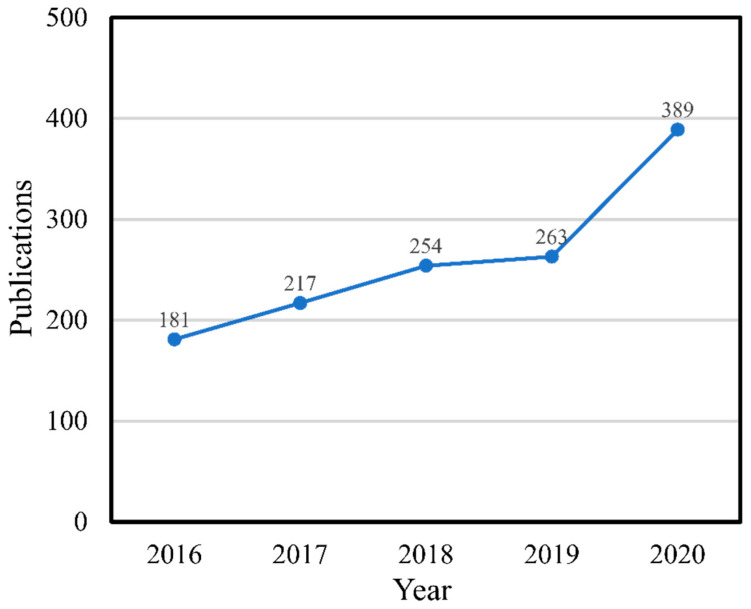
Trend of publications on *Centella asiatica* extraction from 2016–2020 in Scopus.

Extraction is the separation of medicinally active compounds from plant parts using selective solvents through chosen procedures, leaving behind the insoluble compounds. The extracts after solvent removal are obtained in liquid form or as a dry powder consisting of a mixture of compounds. In general, the extraction of plant material is done by washing out the analyte from the matrix into the solvent and diffusion through the cell wall [34]. Over the five years from 2016 to 2020, many papers on the extraction of *C. asiatica* have been published in Scopus, mainly from India (305), China (191), Malaysia (153), Indonesia (80), and Thailand (66). Figure 1 shows that the number of publications on *C. asiatica* extraction has increased during the period, thus proving the interest in this plant. Several methods for preparing *C. asiatica* extracts have been reported, for example, the one developed by Duval [35], who used at least 30 wt.% of an alcoholic solvent in the extraction to obtain a mixture of madecassoside and terminoloside in a refined extract of *C. asiatica*. Kim et al. [36] patented a method to obtain a water-soluble extract of asiaticoside and madecassoside from *C. asiatica*; Loiseau et al. [37] reported a method to obtain an extract consisting of a mixture of madecassoside, asiaticoside, and terminoloside that was more than 75 wt.% relative to the extract total weight, and an extract consisting of a mixture of madecassoside and terminoloside that was more than 95 wt.% pure relative to the total weight of the mixture.

Usually, extraction of *C. asiatica* is done for its phenolic and flavonoid compounds. A wide range of technologies has been used until now to yield a high-quality extract of *C. asiatica* at moderate cost and with a shorter extraction time. Selection of the extraction technique depends on the economic feasibility and suitability of the process for the target compounds. Since the amount of bioactive compounds in *C. asiatica* are relatively small, the extraction method must be chosen carefully to obtain the desired compound from the herb. The types of technique utilized to extract bioactive compounds/essential oil from *C. asiatica* are shown in Table 1.

### 2.1. Maceration

Maceration is a technique in which plant materials are soaked in a solvent at a specific temperature and time [57]. The extract from this method is concentrated using a rotary evaporator to obtain the final solvent-free crude extract. This process softens the plant cells and eventually releases the bioactive compounds from the cells. The solvent used for maceration is based on the study objectives. Maceration is a simple extraction method and has broad applicability. However, it has a long extraction time, high temperature, high mass transfer resistance, low extraction efficiency, and requires a large volume of solvent [57,58].

**Figure 2:**
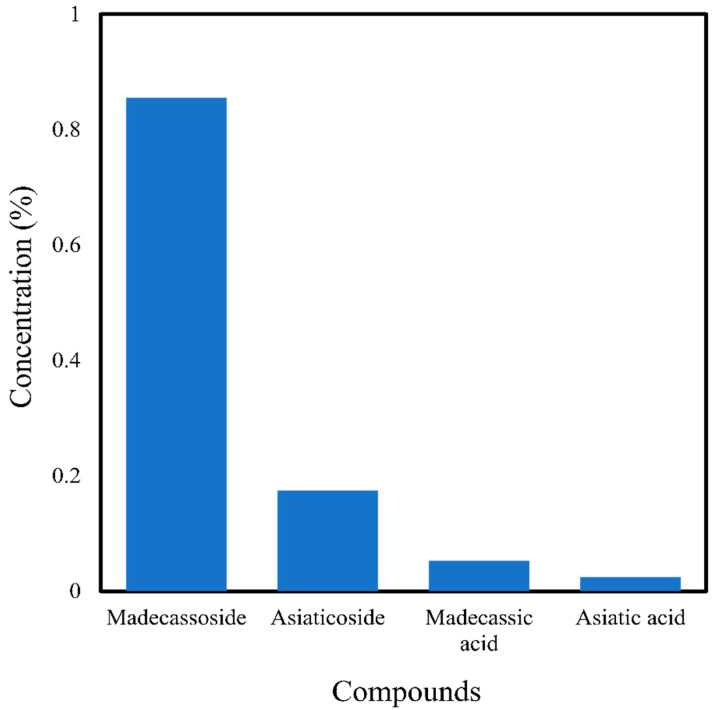
Triterpenoids found in *Centella asiatica* extract using the maceration method of Monton et al. [6].

In the case of *C. asiatica*, organic solvents like ethanol, methanol, or a mixture of alcohol and water are typically used. Aqueous extracts obtained by this technique usually show antioxidant and cytotoxic activity [6,59]. Maceration has also been used in cosmetics manufacturing, in which propylene glycol and water are used as solvents, and the leaves and stalks of *C. asiatica* are extracted for a few days [60]. In a study by Monton et al. [6] using maceration, the highest amounts of madecassoside, asiaticoside, madecassic acid, and asiatic acid (0.855%, 0.174%, 0.053%, and 0.025%, respectively) were extracted at optimal conditions of 60 °C and 120 min extraction time (Figure 2). In a study using cold maceration, Pittella et al. [59] obtained phenolic and flavonoid constituents from *C. asiatica*. Maceration of *C. asiatica* is also able to extract various types of compounds such as triterpenoids, flavonoids, phenolics, saponins, alkaloids, tannins, and carotenoids, primarily based on the solvents used and period of extraction [7,42,43].

### 2.2. Distillation

Distillation is the separation of components at a particular boiling point and condensation. There are two types of distillation used in extraction: steam distillation that is performed by passing dry steam through the plant material, and water distillation in which elevated pressure is used with plants whose essential oil is difficult to extract at a higher temperature [61].

**Figure 3:**
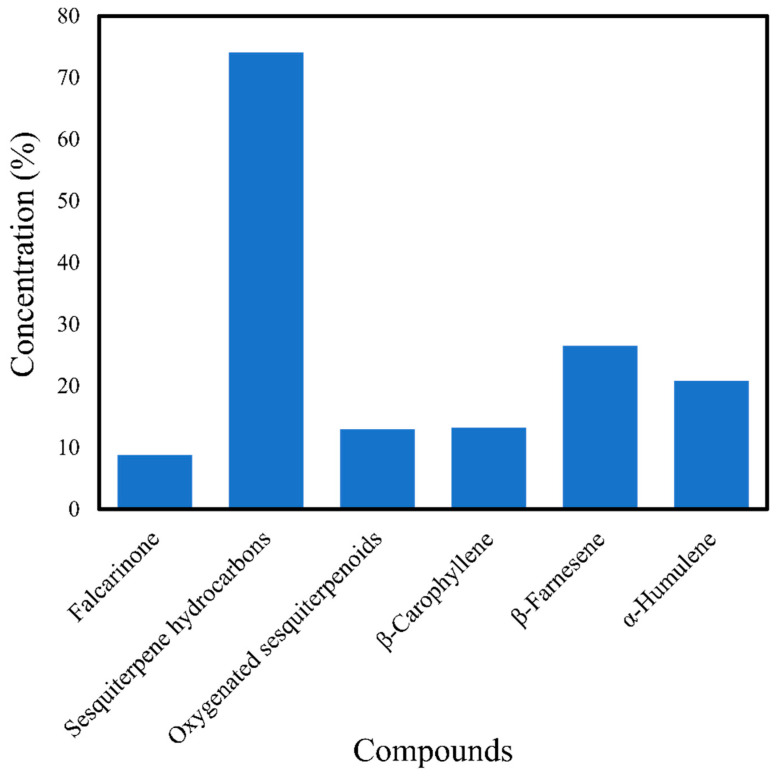
Compounds obtained from *Centella asiatica* using the distillation method of Paudel et al. [32].

Steam distillation using distilled water and vinegar has been used to extract dry and fresh leaves of *C. asiatica*. Steam distillation is an efficient technique for obtaining the best quality of oil, and by employing fresh leaves over dry leaves in extraction, many constituents can be detected [44]. The essential oil of *C. asiatica* obtained by Florczak [44] from steam distillation yielded more sesquiterpenoid hydrocarbons; 43 constituents were identified, representing 98.60% of the composition of the oil. On the other hand, water distillation is an excellent method for extracting caryophyllene and monoterpenoid hydrocarbons from *C. asiatica*; 54 constituents were identified, representing 98.29% of the total composition. However, water distillation utilizes a tremendous amount of water besides consuming a lot of energy and time [62]. A study by Orhan et al. [63] successfully extracted 47 components representing 88.9% of the essential oil. The dominant compound was α-copaene (22.0%), followed by alloaromadendrene (7.6%), β-caryophyllene (7.1%), α-humulene (6.7%), and β-cubebene (5.9%). Paudel et al. [32] discovered that distillation of 85 g of dry *C. asiatica* leaves for 4 h extracted a 0.05% yield of essential oil composed of 33 compounds. The essential oil was rich in sesquiterpene hydrocarbons (74.1%) and oxygenated sesquiterpenoids (13.0%), the most abundant compounds being β-farnesene (26.5%), α-humulene (20.9%), β-caryophyllene (13.3%), and falcarinone (8.8%) (Figure 3).

### 2.3. Soxhlet Extraction

Soxhlet extraction is a technique used to obtain semi-volatile and non-volatile compounds from *C. asiatica* [64]. In this method, the herb is placed in a thimble or porous bag in the Soxhlet rig. The extracting solvent in a round-bottomed flask is boiled at the desired temperature and its vapors are condensed in a condenser. The cooled vapor drips onto the sample of herbs and extracts by contact. When the liquid in the thimble rises to the overflow level, a siphon aspirates the solution into the round-bottomed flask. This cycle is continued for several hours until an adequate phytochemical is acquired. The solvent mixture is then concentrated by a rotary evaporator. Since the sample is frequently exposed to the solvent, and the temperature of the extraction is higher than room temperature, more analytes can be extracted from the sample. Additionally, no filtration is required. The downsides of this method are that it involves a long extraction time, and a large amount of solvent is consumed, which is expensive to dispose of and can cause environmental pollution [64]. In terms of acquiring a volatile compound, the Soxhlet method has been found to be terrible for extraction [44]. The long extraction time and high temperature increase the probability of thermolabile substances being degraded [34]. It has also become an unattractive method for analyzing a high number of samples due to its long extraction time, and samples can only be extracted one at a time for each apparatus.

**Figure 4:**
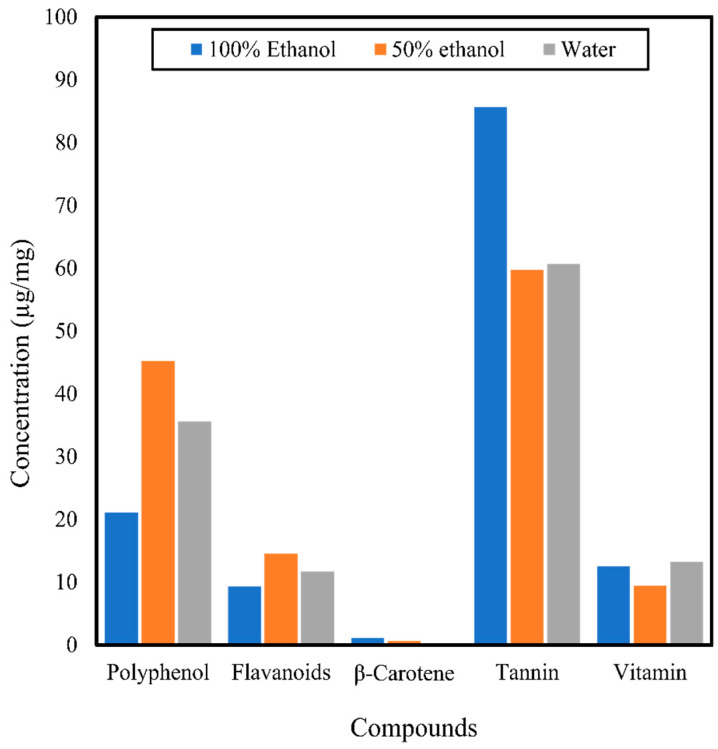
Compounds extracted from *Centella asiatica* using 100% ethanol, 50% ethanol, and water in the Soxhlet extraction method by Rahman et al. [50].

Soxhlet extraction has also been used to obtain the crude extract of *C. asiatica*, which will be later screened for its antimicrobial activity. Several organic solvents such as hexane, chloroform, methanol [17], petroleum ether, acetone [65], and water [11] have been used in the extraction for this purpose. Byakodi et al. [10] discovered that the methanolic extract of *C. asiatica* from the Soxhlet method contained phenols, tannins, flavonoids, terpenoids, saponin, and alkaloids. Another study by Thamarai Selvi et al. [47] found that 500 g of powdered *C. asiatica* subjected to Soxhlet extraction for 8 h using an ethanol to solid ratio of 1:4 resulted in extracts containing saponins, terpenoids, alkaloids, and phenols but no steroids, flavonoids, tannins, proteins, carbohydrates, or glycosides. In a preliminary phytochemical screening of the *C. asiatica* extract, Jayaprakash and Nagarajan [65] discovered the existence of alkaloids, saponins, flavonoids, phenols, steroids, tannins, glycosides, triterpenoids, and terpenoids. The extract also contained 1–8% saponins, 0.1% volatile oils, triterpenic acids (e.g., terminolic acid, brahmic acid, centellic acid, madasiatic acid), and glycosides (e.g., madasiaticoside, brahminoside, centelloside) [60]. Rahman et al. [50] used 100% ethanol, 50% ethanol, and water as solvents for Soxhlet extraction to obtain total polyphenols, flavonoids, β-carotene, tannins, and vitamin C from *C. asiatica*. The study showed that the 50% ethanol extract of *C. asiatica* contained a significantly higher amount of polyphenols and flavonoids while 100% ethanol extracted the highest amount of β-carotene and tannins. On the other hand, the water extract of *C. asiatica* contained more vitamin C than the 50 and 100% ethanol extracts (Figure 4).

### 2.4. Ultrasound-Assisted Extraction

Ultrasound-assisted extraction (UAE) is an extraction method in which ultrasonic waves produce acoustic cavitation in the solvent and cause the disruption of cells. This disruption promotes the release of bioactive compounds and enhances the contact surface area between solid and liquid phases [66,67,68]. Ultrasound causes surface exfoliation, abrasion, and particle disintegration, which increase the mass transfer from the cell cytoplasm to the surrounding solvent [51]. However, frequencies of more than 20 kHz may affect the bioactive compounds through the formation of free radicals [18]. Although this method has a short extraction time and low solvent usage, a minimal sample size is needed to achieve better extraction efficiency [51]. This method is the best approach to recover a high yield of phenolic and flavonoid compounds with the highest antioxidant activity such as betacyanin and anthocyanin, and lipids and protein [69]. It is also highly efficient and causes less destruction of the bioactive compounds since elevated temperature is not used [70]. Thus, it is applicable for extracting thermolabile and unstable compounds [34].

**Figure 5:**
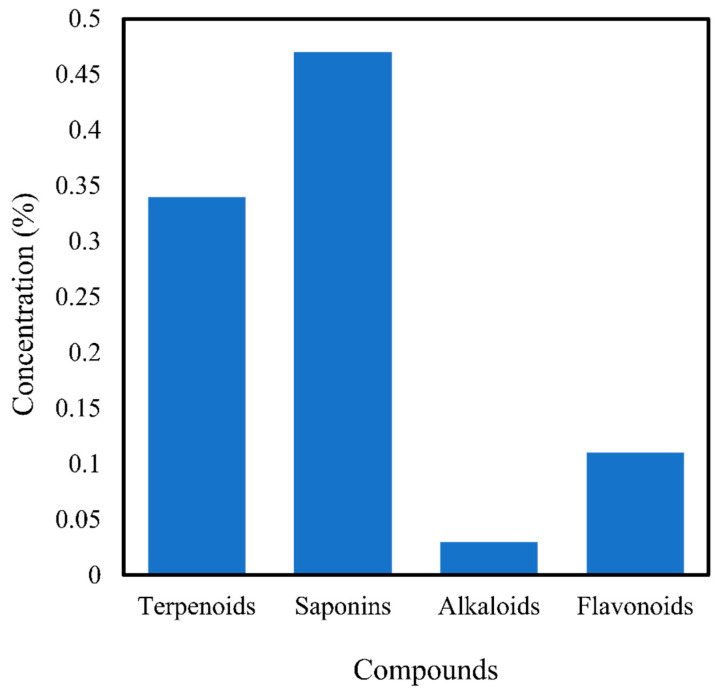
Bioactive compounds extracted using the ultrasonic extraction method of Sellathoroe et al. [52].

Sellathoroe et al. [52] found that UAE gives a higher yield than both Soxhlet and maceration methods for an extract consisting of 0.34% terpenoids, 0.47% saponins, 0.03% alkaloids, and 0.11% flavonoids, as shown in Figure 5 [52]. A study by Nithyanandam et al. [71] demonstrated that UAE is the best extraction method for *C. asiatica* for the significant recovery of antioxidant compounds. Furthermore, the extract contained 79% 1,1-diphenyl-2-picrylhydrazyl (DPPH) scavenging activity, a total phenolic content (TPC) of 1350 mg GAE/100 g DW, and total flavonoid content (TFC) of 599 mg CE/100 g DW, all of which were higher than those obtained using maceration, Soxhlet, and hot-water extraction. This method also extracted 9.24 ± 0.88% of bioactive compounds, which increased to 11.80 ± 0.58% using focused high ultrasound, of which 30.21% was polyphenols [72]. The diffusion and solubility of the solvent were increased by using a 120 kHz ultrasonic wave energy or higher, thus causing more polyphenols to be extracted [72].

Methanol extract of *C. asiatica* leaf has more constituents (alkaloids, terpenoids, glycosides, steroids, tannins, flavonoids, and reducing sugars) than the acetone extract (no terpenoids) and chloroform extract (no tannins). However, UAE using those three solvents is unable to extract saponins [73]. The study by Nithyanandam et al. [71] also showed that a better extract yield was obtained with binary solvent (40:60 ethanol–water) than with pure solvent (100% water and 100% ethanol). Increased yield of TPC and TFC, and DPPH antioxidant activity was proportional to an increase in temperature, but above 45 °C, DPPH scavenging activity decreased drastically while TPC and TFC yield was maintained [71]. The high recovery of phenolic and flavonoid compounds using the UAE technique causes a high percentage of DPPH scavenging activity, which is an important assay to test antioxidant properties. The disadvantage of this technique is the long exposure of the sample to ultrasound activity, which may degrade the yield of asiaticoside and asiatic acid [51].

### 2.5. Microwave Extraction

Microwave extraction is a technique that utilizes fast heating of aqueous samples. The key concept of the method is that the solvent absorbs the microwave energy, which is then transferred in the form of heat to the sample [48]. The energy is transferred into the solvent by the twin mechanism of ionic conduction and dipole rotation [69]. Usually, a solvent such as methanol or water mixture is used for polar compounds and hexane is used for non-polar compounds. Extraction of active compounds from the sample to solvent is influenced predominantly by the temperature and the nature of the solvent.

Contrary to classical heating, microwaves heat the whole sample concurrently. Microwave-assisted extraction (MAE) is a process that operates under higher temperature and in an oxygen-rich environment. It is an excellent technique for reducing the time and solvent consumed to extract target compounds compared to conventional methods [48,56]. This method has a higher extraction rate and produces better products but at a lower cost. Many studies have already proven that it is a feasible option to conventional techniques for many kinds of samples.

**Figure 6:**
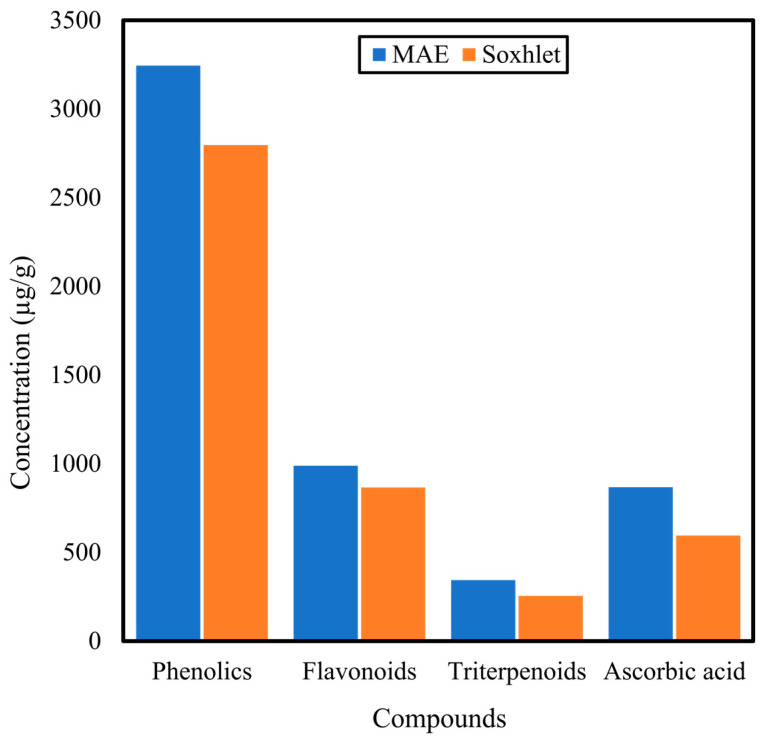
Difference of compound concentrations extracted from *Centella asiatica* using microwave and Soxhlet extraction methods by Sen et al. [54].

Shen et al. [48] found for extraction of *C. asiatica* by MAE that using 90% methanol for 20 min was the optimal condition to extract the highest yield. In another study by Desai et al. [38], the extracts yielded by the MAE method had a 26% increase of TPC and 8% increase of saponin content compared to the traditional solvent extraction. However, the extracts did not exhibit anti-inflammatory activity. A study by Sen et al. [54] using MAE found that it was a much better method for obtaining TPC and triterpenoids than Soxhlet: the extraction time was only 6 min compared to 36 h by Soxhlet, but also the microwave produced 200 times less carbon load. Figure 6 shows the difference in compound concentrations obtained using MAE and Soxhlet extraction. Increasing the microwave power from 170 to 425 W also increased the TPC from 20 to 50%, due to the rapid transfer of electromagnetic energy in the microwave, thus simultaneously heating the plant sample. This situation causes internal stress inside the plant and eventually bursts the cell wall, releasing its contents [54].

Regardless of the benefits, the interaction between the sample and oxygen during MAE operated at a very high temperature may cause a destructive effect on the desired bioactive compounds, especially if they are oxygen- and heat-sensitive [39,74]. Hence, vacuum microwave-assisted extraction (VMAE) has been developed as an option to acquire that type of bioactive compounds [39]. It is a method in which the boiling temperature of an extraction solvent is lowered, consequently reducing the extraction temperature. The pressure is also reduced so that less oxygen is present for the unfavorable process of oxidation. Fresh and dried leaves of *C. asiatica* were treated with VMAE to extract triterpene saponins and phenolics at various pressures. It was found that the fresh leaves of *C. asiatica* contained the maximum triterpene saponins when VMAE was performed at 60 kPa, while the maximum TPC was obtained when extraction was carried out in atmospheric conditions. On the other hand, the dried leaves contained the highest triterpene saponins and TPC when they were extracted at atmospheric pressure: the temperature was higher, which caused greater diffusivity of bioactive compounds and resulted in a more elevated amount of triterpene saponins and TPC. Otherwise, at 20 kPa, the temperature of extraction was reduced (~40 °C), thus minimizing diffusion of the bioactive compounds [39].

MAE has also been improved by using solvent-free microwave extraction (SFME). The method utilized a vacuum and stirrer during extraction and operated at 300 W for 15 min to extract 158 µg/mL of asiaticoside from *C. asiatica*. Solventless extraction saves the use of organic solvents, and the extraction is performed in a shorter time due to direct heat radiation to the samples, which results in rapid extraction [55].

A study by Wang et al. [56] on enzymatic pretreatment and microwave extraction (EPME), combining enzymolysis and microwave extraction, showed that it has high extraction efficiency, takes less time, and is an environmentally friendly way of extracting asiaticoside from *C. asiatica*. However, the downside of this technique is that it is expensive because enzymes are used, and the complexity of industrializing EPME would restrict further application.

### 2.6. Subcritical Water Extraction

Subcritical water extraction is a method that modifies the physical properties of water under high pressure and increases the temperature above its boiling point (up to 374 °C) to maintain the water in its liquid state, thus improving it as an extraction solvent. Subcritical water extraction is an efficient, harmless, and eco-friendly method for extracting polar compounds from samples, which is an excellent substitute for conventional organic solvent extraction methods. Furthermore, this method requires less extraction time and solvent, but acquires a higher quality of extracts [40].

**Figure 7:**
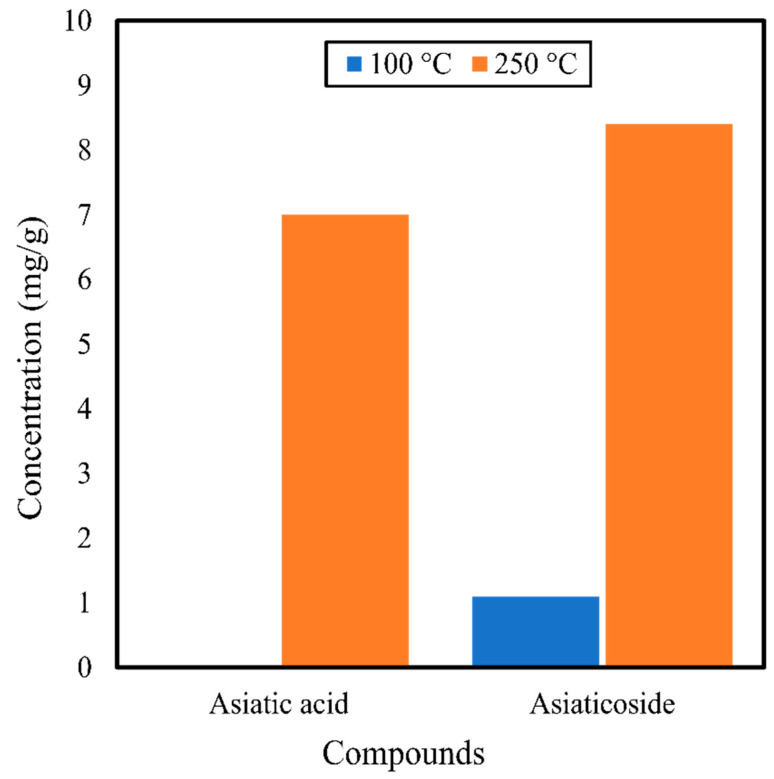
Compounds obtained from *Centella asiatica* using the subcritical water extraction method by Kim et al. [40] at 100 and 250 °C.

There are not many studies on the compounds, in vitro analysis, or in vivo analysis of *C. asiatica* obtained by subcritical water extraction. A study by Kim et al. [40] on the extraction of *C. asiatica* with the subcritical water extraction method demonstrated a rise in the concentration of asiatic acid from 0 to 7.0 mg/g, and in that of asiaticoside from 1.1 to 8.4 mg/g, when the temperature was increased from 100 to 250 °C (Figure 7). The enhanced solubility and enhanced transport properties of subcritical water at a higher temperature may be responsible for the higher extraction yields of asiatic acid and asiaticoside. On the other hand, the outcome was not heavily reliant on the pressure of the extraction process because there was little increment in the yield of asiaticoside (from 4.6 to 8.1 mg/g) or asiatic acid (from 2.4 to 3.4 mg/g) on increasing the pressure from 10 to 40 MPa. Regarding the effect of pressure on the extraction, there were not many changes toward the polarity of the subcritical water. Hence, subcritical water pressure lacks influence on the extraction yield.

## 3. Antimicrobial Activity

Plant-based antimicrobials are often studied and used in medicine as they have fewer side effects compared to synthetic antimicrobials. *C. asiatica* is one of the plants that have antimicrobial activity against many types of bacteria and fungi since the triterpenoids in *C. asiatica* can be regarded as phytoanticipins due to their selective cytotoxicity and protective role in preventing infections from a pathogen [75,76]. Flavonoids and tannins are known for their antimicrobial activity [65]. An antimicrobial susceptibility test is essential to determine the efficiency of a plant extract against the growth of microbes. Besides targeting the desired compounds in *C. asiatica*, the extracts obtained from selected extraction methods were further analyzed for their antimicrobial properties. Many studies have been done on the activity of *C. asiatica* extracts against pathogens. Still, they are hard to compare depending on the type of extraction method, solvents, strains of microbes, and antimicrobial test methods used [77].

### 3.1. In Vitro Studies

The disc diffusion method is widely used as it is suitable for preliminary testing for screening the antimicrobial activity of plant extracts [78]. The susceptibility of microbes to the plant extract is determined by inoculating microbial suspension onto the medium agar surface and swabbing it evenly all over the agar surface with a cotton swab. The filter paper discs are dipped with plant extract and incubated for 24–48 h. The inhibition zones are observed, and their diameter measured, indicated by a clear area around the filter paper disc. A clear zone of inhibition reveals the microbes’ vulnerability to an extract, while the absence of such a zone shows the microbes’ resistance to the extract. This method is a low-cost and straightforward technique to determine the efficiency of a plant extract in inhibiting the growth of microbes [79]. For the agar well diffusion method, the agar is diffused with the plant extract before it is solidified, and each plate is inoculated with microbial culture and spread evenly with a sterile bent glass rod. The agar medium is cut with a sterile cork borer, and different concentrations of plant extract solution are loaded by micropipette into the agar well. Plates are incubated for 24–48 h before the zones of inhibition are observed. There is also the determination of the minimum inhibitory concentration (MIC) by the microdilution method using 96-well microtitration plates, the hole plate diffusion method, the two-fold method in a microtiter plate, the Versa Max Tunable microplate reader, and particular MIC methods such as the tetrazolium microplate method, liquid dilution method, and serial dilution method [80]. In terms of MIC, concentrations of extracts are varied in the preferred antimicrobial test to determine the lowest concentration of extract that effectively prevents microbial growth [81].

A study by Zheng et al. [82] using an extract of *C. asiatica* obtained by the maceration method found that at 2 mg/mL, it inhibited the growth of *Helicobacter pylori* by the agar well diffusion method; the MIC against the strains tested ranged from 0.125 to 8 mg/mL. Methanol extract obtained by the maceration method showed a broad spectrum of antimicrobial activity using the agar diffusion method against both Gram-positive and -negative bacteria, with the zones of inhibition ranging from 9 to 29 mm and the MIC ranging from 1.25 to >10 mg/mL [76]. However, while maceration of *C. asiatica* using methanol produced an extract that was unable to inhibit *Escherichia coli* [83,84], positive inhibition was achieved when using ethanol as the solvent [85,86].

Vadlapudi et al. [17] used the agar well diffusion method to determine the antimicrobial activity of *C. asiatica* from inhibition zones and MIC values. Methanol extract of *C. asiatica* from Soxhlet extraction was able to inhibit *Aspergillus niger* (14–19 mm), *Fusarium oxysporum* (13–14 mm), *Xanthomonas campestris* (10–13 mm), *Lactobacillus acidophilus* (11–13 mm), *Pseudomonas marginalis* (10–25 mm), *Pseudomonas syringae* (18–22 mm), *Staphylococcus salivarius* (13–177 mm), and *Staphylococcus aureus* (9–12 mm), but exhibited no activity toward *Penicillium expansum*, *Pseudomonas aeruginosa*, or *Staphylococcus mutans*. The MIC found were 0–155 mg/mL. In another study, aqueous extract from maceration exhibited better antimicrobial activity toward *Bacillus cereus*, *E. coli*, *P. aeruginosa*, *S. aureus*, and *S. mutans* by the well diffusion method compared to the ethanol extract [87]. The MIC of the aqueous extract was 25 mg/mL for *B. cereus* (3.00 ± 0.00 mm), *E. coli* (6.00 ± 0.00 mm), and *S. mutans* (3.00 ± 0.00 mm). On the other hand, the MIC of the aqueous extract was 50 mg/mL for *P. aeruginosa* (6.33 ± 0.58 mm) and *S. aureus* (10.0 ± 0.00 mm).

**Figure 8:**
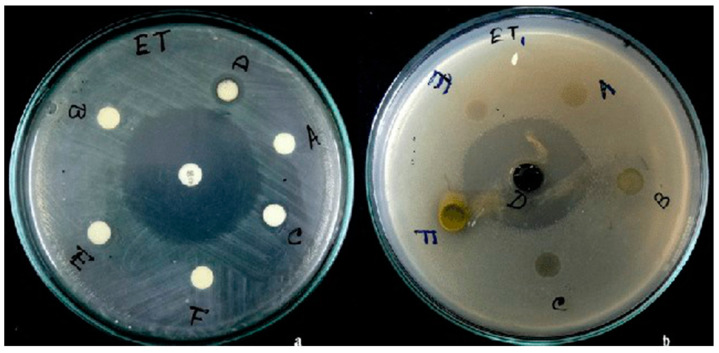
Inhibition of *Edwardsiella tarda* by (**a**) agar disc diffusion and (**b**) agar overlay well diffusion assays. A: aqueous control; B: aqueous test; C: chloroform control; D: chloroform test; E: methanol control; F: methanol test; C 30: chloramphenicol, 30 µg/disc; 10 μL/disc and 50 μL/well used for agar disc diffusion and agar overlay well diffusion assays, respectively. Figure and caption reused from Purkait et al. [88]. Used under the Creative Commons License (http://creativecommons.org/licenses/by/4.0/ (accessed on 26 February 2021)).

**Figure 9:**
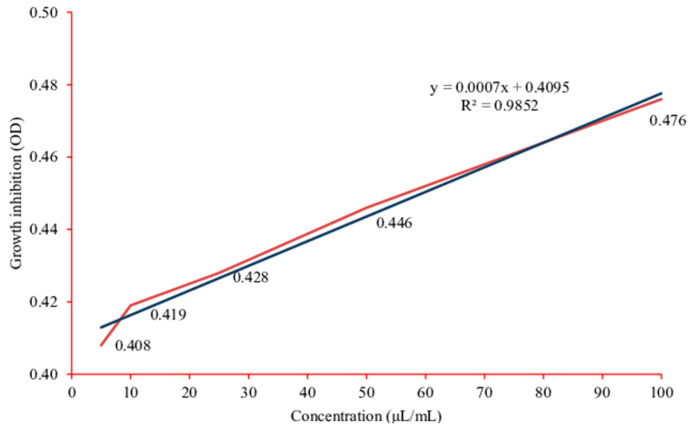
Growth inhibition of *Edwardsiella tarda* by crude *Centella asiatica* chloroform extract in the broth dilution assay. OD: optical density. Figure and caption reused from Purkait et al. [88]. Used under the Creative Commons License (http://creativecommons.org/licenses/by/4.0/ (accessed on 26 February 2021)).

Purkait et al. [88] studied the efficacy of aqueous, methanol, and chloroform extracts of *C. asiatica* against the fish pathogenic bacteria *Aeromonas hydrophila* and *Edwardsiella tarda* using agar disc diffusion, agar overlay well diffusion, and broth dilution assays. All types of *C. asiatica* extract failed to inhibit *A. hydrophila*. On the other hand, *E. tarda* was inhibited by chloroform extract (11.25 ± 0.35 mm) using the agar disc diffusion assay. In the agar overlay well diffusion assay, 50 μL of chloroform and methanol extracts of *C. asiatica* inhibited *E. tarda* (30.50 ± 6.40 and 7.50 ± 0.70 mm, respectively) (Figure 8). The crude chloroform extract of *C. asiatica* using agar overlay well-diffusion assay produced the largest zone of inhibition (30.50 ± 6.40 mm), which was comparable to those of the chloramphenicol (40.75 ± 1.76 mm). In the broth dilution assay, increasing the concentration of crude chloroform *C. asiatica* extract resulted in increased inhibition of *E. tarda* growth (Figure 9). It was concluded that the chloroform extract of *C. asiatica* is the most effective to control *E. tarda* infection, especially in aquaculture.

Methanol extract of *C. asiatica* from Soxhlet extraction was found to inhibit various types of fungi (e.g., *Aspergillus niger*, *Alternaria alternata*, and *F. oxysporum*) and bacteria (e.g., *P. syringae*, *S. aureus*, and *Bacillus subtilis*) [17,65,89], but petroleum ether extract did not show any antimicrobial activity against the microbes tested [65]. Byakodi et al. [10] also found that a methanolic extract obtained by the Soxhlet method exhibited antimicrobial activity against Gram-positive and -negative strains of bacteria. Ethanolic extract obtained by the Soxhlet method was found to exhibit better antimicrobial activity toward *E. coli* (extended-spectrum β-lactamase-producing (ESBL) and carbapenem-resistant strains), *Klebsiella pneumoniae* (carbapenem-resistant strains), and *P. aeruginosa* (carbapenem-resistant strains) in contrast to the petroleum ether extract, which was only efficient in inhibiting *K. pneumoniae* (ESBL strains). However, both extracts were unable to inhibit the growth of *Acinetobacter baumannii* [90]. ESBL-producing Enterobacteriaceae cause infections in the urinary tract, and drug-resistant infections were found to cause 9000 deaths, of which 600 were due to carbapenem-resistant *K. pneumoniae* and *E. coli* [90,91]. Therefore, new antibacterial agents are needed to counter the pathogen, and the extract of *C. asiatica* could be one of them.

Mostly, extraction of *C. asiatica* to determine the antimicrobial activity of the extract is done by a conventional method such as maceration or Soxhlet. Still, several studies have used methods such as UAE and MAE: extracts of *C. asiatica* obtained using these methods also showed positive inhibition of the growth of studied bacteria. UAE of *C. asiatica* produced an extract that could inhibit *P. aeruginosa* (6.5 mm zone of inhibition) and *E. coli* (8.5 mm) [52]. Similarly, Sellathoroe et al. [52] found that the extract from UAE was the best for inhibiting the growth of Gram-negative bacteria. At a concentration of 100 µg/mL, methanol extract of *C. asiatica* leaves obtained by UAE showed the largest zone of inhibition against *E. coli* (30 mm), followed by *B. cereus* (29 mm), *P. aeruginosa*, and *S. aureus* (both 28 mm) [73]. Asiaticoside and asiatic acid are prominent bioactive compounds in *C. asiatica* leaves; these compounds display efficacy against Gram-negative bacteria such as *S. aureus* and *E. coli* [92].

In general, the methanolic extract of *C. asiatica* shows a better inhibitory effect than acetone, chloroform, and water extracts [65,73,77]. The extract is also rich in compounds like terpenoids, saponins, phenols, flavonoids, and tannins that consequently contribute to better antimicrobial activity [10]. Antimicrobial activity tests also indicate that *C. asiatica* is a good source of an antimicrobial agent against a wide range of microbes. Details of the effectiveness of *C. asiatica* extracts obtained by several different extraction methods toward various types of microbes are shown in Table 2.

### 3.2. In Vivo Studies

Although many studies have been done on the antimicrobial properties of *C. asiatica* in vitro, they are not a complete test to summarize the antimicrobial activity and analogize the way the extract will act in vivo. There are other circumstances such as first-pass metabolism, microbial defense, drug resistance, and conditions of the patient’s pathology that will also influence the effectiveness of the test [79]. Few antimicrobial studies are done in vivo due to their complexity and expense; not only does the activity against the microbes need to be assessed, but there is also a concern regarding possible allergic reactions and mammalian cell toxicity [80]. Most of the studies of *C. asiatica* in animal models have focused on wound healing since the plant is famous for its antioxidant and anti-inflammatory activity [21,41,99,100].

**Figure 10:**
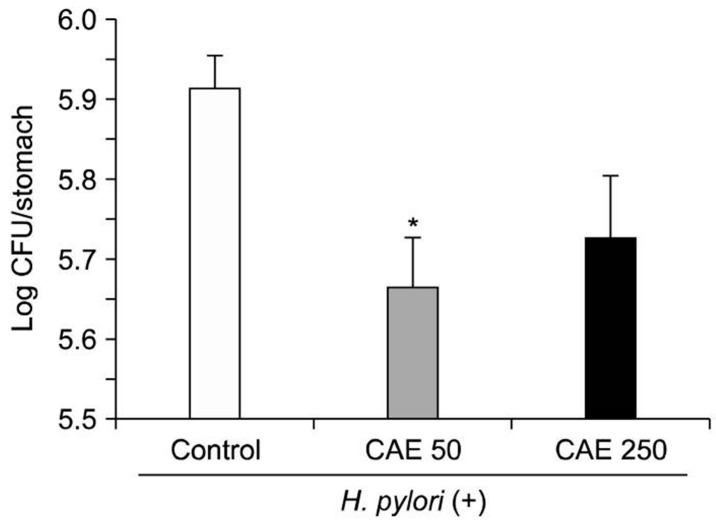
Effects of *Centella asiatica* leaf extract (CAE) against *Helicobacter pylori* colonization in C57BL/6 mice. CAE was administered orally at 50 and 250 mg/kg, once daily for three weeks. The results are expressed as mean ± SEM (*n* = 10). * *p* < 0.05 compared with the control. Figure and caption reused from Zheng et al. [82]. Used under the Creative Commons License (http://creativecommons.org/licenses/by/4.0/ (accessed on 26 February 2021)).

Figure 10 shows the effectiveness in vivo of *C. asiatica* extracts obtained using maceration in reducing *H. pylori* gastric mucosal colonization in a C57BL/6 mouse model. The optimum concentration was 50 mg/kg after oral administration once daily for three weeks [82].

There was also an in vivo study of *C. asiatica* as an anti-acne gel, in which the antibacterial effect on *Propionibacterium acne* was observed and measured through sebum secretion. Clinical assessment using a skin analysis tool on 12 volunteers showed decreased symptoms of inflammation, the number of papules, nodules, and pustules, and shifts in sebum levels [101].

The aqueous extract of *C. asiatica* was used to control the fish disease columnaris in Nile tilapia caused by the bacterium *Flavobacterium columnare*. Fish mortality was decreased depending on the doses used (0, 20, 40, and 60 mg/L) and at 100 mg/L of extract, no mortality or adverse effects were found in the infected fish [102].

## 4. Safety and Toxicology

Toxicity is the level of adverse health effects on living organisms from the interaction between living cells and selective toxicants. A toxicity test is crucial to ensure the safety and efficacy of plant extracts. This can be done using the brine shrimp lethality assay (BSLA) using different concentrations of crude plant extracts [103,104]. Brine shrimp (*Artemia salina*) have been used in over 90% of studies using *Artemia* as a test organism since it is simple, inexpensive, rapid, convenient, and requires a small amount of test material. LD50 is the concentration required to obtain the death of 50% of the test population with the BSLA. An extract is considered toxic if the LD50 is less than 1000 µg/mL, weakly toxic if 500 to 1000 µg/mL, and non-toxic if the LD50 is more than 1000 µg/mL [105].

It was found that *C. asiatica* has cytotoxic activity from 500 µg/mL to above 1000 µg/mL, suggesting that it is weakly or insignificantly cytotoxic [103]. Ethanolic extract of *C. asiatica* was discovered to have an LD50 of more than 1000 µg/mL at three different concentrations of *C. asiatica* (100, 500, and 1000 µg/mL) against 28.7 µg/mL potassium dichromate [106]. All concentrations posed an insignificant toxicity level after 24 h of exposure. The mortality rate of brine shrimp was 3.33% at a concentration of 10 µg/mL, 20.00% at 100 µg/mL, and 40.00% at 1000 µg/mL of *C. asiatica* ethanolic extract, after 24 h of exposure [107]. Calculation of LD50 was 1926 µg/mL against the standard drug, etoposide. The maximum toxic concentration was 60,822 µg/mL, and the minimum limit of toxic concentration was 606 µg/mL, showing a lower cytotoxicity level of *C. asiatica* [103]. Selvi et al. [108] reported an LD50 for aqueous and chloroform extracts of *C. asiatica* of 840 and 765 µg/mL, respectively, also showing low toxicity of *C. asiatica*. The type of solvent used to obtain extracts influenced the LD50 results because different solvents have different extraction potential for toxicity screening. Furthermore, some solvents are better than others for extracting bioactive compounds from plants that might be toxic [103]. Preparation of stock solutions and dilution factors of sample extracts can also affect the concentrations of sample solutions, thus directly influencing the toxicity results for sample extracts during biological screening [105].

The efficacy, performance, and safety of *C. asiatica* have been witnessed and widely applied in traditional Indian, Asian, and Chinese medicines, herbs or food and beverages, and pharmaceutical products [109]. A report by the Cosmetic Review Ingredient [60] expert panel confirmed the safety of various *C. asiatica* extracts in the current practices of use, based on the reported research including limited data on in vitro human cell cultures and oral administration in human studies. According to the World Health Organization (WHO), the recommended dosage for oral intake of *C. asiatica* is 1.00 to 2.00 g per day for scar surface or wound healing. In terms of tea or juice preparation using the dried plant, the dosage is 0.33–0.68 g per meal [1,42]. To date, no toxic effects from *C. asiatica* intake have been reported by the WHO [42]. *C. asiatica* extracts and asiatic acid were orally administered in an experimental hamster and rabbit model. No toxic effect was observed after intake of 1.0 mg/kg of asiatic acid [59] or 1 mg/kg of asiaticoside [110]. In fact, administering asiaticoside at 1.00 g/kg of the patient’s body weight has been demonstrated to be non-toxic in the oral application of *C. asiatica* extract [1]. Acute oral administration of 1 g/kg body weight of an ethanolic 50% extract and alcoholic extracts of *C. asiatica* also showed no toxicity at doses of 350 mg/kg when given to rats [111].

The European Medicine Agency has reported clinical studies on the effects of *C. asiatica* on chronic venous insufficiency (CVI), periodontitis, psoriasis, ulcer cicatrization, burn recovery, anxiety, and atherosclerosis. Clinical studies showed that *C. asiatica* improves microcirculation and leg volume associated with decreased edema and symptoms. The safety profile of *C. asiatica* extracts appears satisfactory and tolerable as it is emerging from clinical studies in patients affected by CVI and from its use in products on the market. Clinical trials have reported no drug-related serious adverse events. The recommended doses for non-toxic nature with no or infrequent adverse side effects are 60–180 mg daily. However, at a higher dose, there may be occasional burning pain or skin allergy following injection or topical application. In addition, gastric complaints and nausea have occasionally been reported following oral administration of *C. asiatica* extract [32,110]. In general, there are no reasons for concerns relating to safety, and the tolerability of oral *C. asiatica* preparations was good in all studies. No adverse events from pharmacovigilance data are known [111].

## 5. Conclusions

Several methods have been used to extract compounds from *C. asiatica*. The efficiency of extraction is based on the extraction method, extraction solvent, and extraction time. The desired compounds extracted also influence the choice of extraction method aside from cost and availability. Among the applications, the antimicrobial action of *C. asiatica* has been widely studied, mostly in extracts obtained conventionally. To date, very few microbial species have been tested using *C. asiatica* extracts obtained by modern extraction techniques. Thus, more studies are necessary for these extracts to determine their effect on microorganisms. The modern extraction techniques also seem to be more promising for obtaining antimicrobial compounds in terms of cost, time, and better efficacy toward certain microbes compared to conventional techniques. In particular, solventless extraction hinders the possible retention of the chemical solvent in the extract. Therefore, the extracts obtained from these modern techniques are worthy as antimicrobial agents. Both in vitro and in vivo studies have shown that *C. asiatica* possesses antimicrobial activity, although there have been few in vivo studies due to their complexity. Nevertheless, the extracts have the potential to be used in the medicinal, cosmeceutical, and food sectors.

## Figures and Tables

**Table 1:** Methods for the extraction of compounds in *C. asiatica.*

Extraction Method	Extraction Time	Sample Type	Solvent Type	Sample to Solvent Ratio (g/mL)	Additional Information	Compounds Extracted	References
Maceration	6 h	Dried	90% methanol	10:100	66 °C	Phenolics, saponins	[38]
	24 h	Fresh	Ethanol	5:25	Room temperature	Saponins	[39]
		Dried		0.5:25			
	5 h	Dried	Water, ethanol, methanol	3:100	Solvent boiling point	Asiaticoside, asiatic acid	[40]
	120 min	Dried	95% ethanol	20:100	60 °C	Madecassoside, asiaticoside, asiatic acid, madecassic acid	[6]
	30–90 min	Dried	Ethanol–water	1:20	30–60 °C	Polyphenols, carotenoids	[16]
	72 h	Dried	Distilled water	100:1000	-	Gluconic acid, ferulic acid, kaempferol, chlorogenic acid, asiatic acid	[41]
	24 h	Fresh/dried	80% ethanol–water	1:20	Room temperature	Phenolics, triterpene saponins	[42]
	-	Dried	Methanol, petroleum ether, chloroform	-	60–80 °C	Triterpenoids, saponins, tannins, flavonoids	[7]
	24 h	Powder	Methanol	2:100	Room temperature	Valine, triparanol, butamben, neuraminic acid, aesculin, esculetin, famciclovir, isocitretin, rhoifoline, gentiopicrin, urocortisone, pelargonic acid, gabapentin, ivermectin, sarmentoside, khivorin	[43]
Distillation	75 min	Dried	Xylene	0.4:100	-	Essential oil, α-caryophyllene, germacrene D	[44]
	3 h	Fresh	Water	-	-	Essential oil	[45]
	4 h	Dried	Water	-	-	Essential oil	[32]
Soxhlet	12–24 h	Dried	Methanol	-	-	Phenolics, flavonoids, ascorbic acid	[46]
	8 h	Dried	Ethanol	500:125	-	Saponins, terpenoids, alkaloids, and phenols but no steroids, flavonoids, tannins, proteins, carbohydrates, or glycosides	[47]
	1 h	Dried	Methanol–water (9:1)	2:50	-	Asiatic acid, asiaticoside, madecassoside	[48]
	8 h	Dried	Methanol	1:100	-	Asiaticoside, madecassic acid, madecassoside, asiatic acid	[49]
	6 h	Dried	Methanol	-	60 °C	Alkaloids, phenols, tannins, flavonoids, terpenoids, and saponins	[10]
	-	Dried	Ethanol–water (1:1)	1:10	45 ± 2 °C	Total polyphenols, flavonoids, β-carotene, tannins, and vitamin C	[50]
Ultrasound-assisted extraction (UAE)	1 h	Dried	Methanol–water (9:1)	2:50	-	Asiatic acid, asiaticoside, madecassoside	[48]
	20 min	Dried	Water	0.6:50	125 W	Asiatic acid	[51]
	5 h	Dried	Ethyl acetate–water (99:1)	6:150	Frequency: 40 kHz; temperature: 70 °C; power: 216 W	Alkaloids, flavonoids, saponins, terpenoids	[52]
	3 × 10 min	Dried	Methanol–water (9:1)	1:10	-	Asiatic acid, asiaticoside, madecassoside, madecassic acid	[53]
Microwave-assisted extraction (MAE)	20 min	Dried	Methanol–water (9:1)	1:25	-	Asiatic acid, asiaticoside, madecassoside	[48]
	6 min	Dried	Ethanol	1:25	Microwave power: 50%; 40%	Phenolics, triterpenoids Flavonoids	[54]
	5 min with 2 min pauses	Dried	Methanol–water (9:1)	10:100	Microwave power: 100%	Phenolics, saponins	[38]
	20 min	Fresh	Ethanol	10:50	Atmospheric	Triterpene saponins, TPC	[39]
		Dried		1:50			
Vacuum microwave-assisted extraction (VMAE)	20 min	Fresh	Ethanol	10:50	20, 40, 60 kPa	Triterpene saponins	[39]
		Dried		1:50			
Solvent-free microwave extraction (SFME)	15 min	Fresh	-	-	Microwave power: 300 W	Asiaticoside	[55]
Enzymatic pretreatment microwave extraction (EPME)	110 s	Dried	3% cellulase solution	3:108	Enzymolysis 30 min, 45 °C	Asiaticoside	[56]
Subcritical water extraction	5 h	Dried	Deionized water	-	250 °C, 40 MPa	Asiatic acid, asiaticoside	[40]

**Table 2:** Antimicrobial activity of *C. asiatica* extracts obtained by various methods.

Extraction Method	Solvent	Antimicrobial Method	Microbes	Effect	References
Maceration	Methanol, water	Open hole diffusion, 2-fold dilution method	*Bacillus subtilis*	+	[83]
			*Escherichia coli*	−	
			*Aeromonas hydrophila*	−	
			*Citrobacter freundii*	−	
Maceration	Ethanol	Agar diffusion	*Bacillus cereus*	+	[93]
			*Listeria monocytogenes*	+	
Maceration	Ethanol	Disc diffusion	*Escherichia coli*	+	[85]
			*Bacillus subtilis*	+	
			*Vibrio cholerae*	+	
			*Shigella sonnei*	+	
			*Bacillus cereus*	−	
			*Shigella dysenteriae*	−	
			*Staphylococcus aureus*	−	
			*Salmonella paratyphi*	+	
Maceration	Ethanol	Disc diffusion	*Staphylococcus aureus*	+	[92]
Maceration	Water, methanol	Disc diffusion, agar well diffusion	*Aeromonas hydrophila*	−	[88]
			*Edwardsiella tarda*	−	
	Chloroform	Broth dilution, agar well diffusion, disc diffusion	*Aeromonas hydrophila*	+	
			*Edwardsiella tarda*	+	
Maceration	Water	Agar diffusion, Disc diffusion	*Salmonella enterica*	−	[94]
			*Shigella flexneri*	−	
			*Escherichia coli*	−	
			*Enterobacter cloacae*	−	
Maceration	Ethanol	Agar well diffusion	*Helicobacter pylori*	+	[82]
Maceration	Methanol	Micro broth dilution	*Mycobacterium* sp.	+	[84]
			*Staphylococcus aureus*	+	
			*Bacillus subtilis*	+	
			*Aspergillus niger*	+	
			*Candida albicans*	+	
			*Escherichia coli*	−	
Maceration	Acetone	Micro broth dilution	*Bacillus cereus*	+	[77]
			*Serratia* sp.	−	
			*Rhodotorula mucilaginosa*	−	
			*Aspergillus flavus*	−	
			*Penicillium citrinum*	−	
	Methanol		*Bacillus cereus*	+	
			*Serratia* sp.	+	
			*Rhodotorula mucilaginosa*	+	
			*Aspergillus flavus*	+	
			*Penicillium citrinum*	+	
	Ethanol		*Bacillus cereus*	+	
			*Serratia* sp.	−	
			*Rhodotorula mucilaginosa*	+	
			*Aspergillus flavus*	+	
			*Penicillium citrinum*	+	
Maceration	Dichloro-methane:methanol	Disc diffusion, micro broth dilution	*Escherichia coli*	+	[81]
			*Salmonella typhi*	+	
			*Bacillus subtilis*	+	
			*Staphylococcus aureus*	+	
			*Shigella sonnei*	+	
Maceration	Ethanol aqueous	Disc diffusion, agar dilution	*Staphylococcus aureus*	+	[42]
Maceration	Methanol, acetone, ethyl acetate	Agar diffusion, microplate dilution assay	*Pseudomonas aeruginosa*	+	[76]
			*Staphylococcus aureus*	+	
			*Streptococcus agalactiae*	+	
			*Bacillus cereus*	+	
			*Enterococcus hirae*	+	
			*Enterococcus faecalis* (clinical isolate)	+	
			*Enterococcus gallinarum*	+	
			*Escherichia coli*	+	
Maceration	Aqueous	Disc diffusion	*Streptococcus pyogenes*	−	[86]
			*Pseudomonas aeruginosa*	−	
			*Escherichia coli*	+	
			*Staphylococcus aureus*	+	
			*Staphylococcus albus*	+	
			*Streptococcus pneumoniae*	+	
			*Candida albicans*	−	
			*Microsporum boulardii*	−	
			*Aspergillus niger*	+	
			*Aspergillus flavus*	+	
	Aqueous	Open hole diffusion	*Streptococcus pneumoniae*	−	
			*Streptococcus pyogenes*	−	
			*Pseudomonas aeruginosa*	+	
			*Escherichia coli*	+	
			*Staphylococcus aureus*	+	
			*Staphylococcus albus*	+	
	Chloroform	Disc diffusion	*Escherichia coli*	+	
			*Staphylococcus aureus*	+	
			*Staphylococcus albus*	+	
			*Pseudomonas aeruginosa*	+	
			*Streptococcus pyogenesis*	+	
			*Streptococcus pneumoniae*	+	
Soxhlet	Ethanol, methanol	Disc diffusion	*Aspergillus niger*	+	[89]
			*Bacillus subtilis*	+	
Soxhlet	Water	Disc diffusion	*Escherichia coli*	+	[11]
			*Klebsiella pneumoniae*	+	
			*Staphylococcus aureus*	+	
			*Streptococcus pyogenes*	+	
Soxhlet	Aqueous	Agar well diffusion	*Escherichia coli*	+	[95]
			*Staphylococcus aureus*	+	
			*Bacillus megaterium*	+	
			*Vibrio parahaemolyticus*	+	
			*Vibrio mimicus*	+	
			*Shigella boydii*	+	
			*Bacillus cereus*	+	
			*Bacillus subtilis*	+	
			*Shigella dysenteriae*	+	
			*Salmonella typhi*	+	
			*Salmonella* Paratyphi	+	
			*Pseudomonas aeruginosa*	+	
			*Escherichia coli*	+	
			*Sarcina lutea*	+	
			*Staphylococcus aureus*	+	
Soxhlet	Methanol	Disc diffusion	Methicillin-resistant *Staphylococcus aureus* (MRSA)	+	[96]
			*Staphylococcus aureus*	+	
			*Klebsiella pneumoniae*	−	
			*Pseudomonas aeruginosa*	−	
			*Escherichia coli*	−	
Soxhlet	Methanol	Agar well diffusion	*Micrococcus luteus*	+	[10]
			*Staphylococcus aureus*	+	
			*Bacillus subtilis*	+	
			*Bacillus cereus*	+	
			*Escherichia coli*	+	
			*Pseudomonas aeruginosa*	+	
			*Zymomonas mobilis*	+	
		Disc diffusion	*Aspergillus niger*	+	
			*Aspergillus sydouri*	+	
			*Trichoderma reesei*	+	
Soxhlet	Methanol	Agar well diffusion	*Aspergillus niger*	+	[17]
			*Penicillium expansum*	−	
			*Fusarium oxysporum*	+	
			*Xanthomonas campestris*	+	
			*Lactobacillus acidophilus*	+	
			*Pseudomonas marginalis*	+	
			*Pseudomonas syringae*	+	
			*Pseudomonas aeruginosa*	−	
			*Streptococcus mutans*	−	
			*Streptococcus salivarius*	+	
			*Staphylococcus aureus*	+	
Soxhlet	Methanol	Disc diffusion	*Proteus mirabilis*	+	[65]
			*Streptococcus faecalis*	+	
			*Streptococcus pyogenes*	+	
			*Escherichia coli*	+	
			*Fusarium oxysporum*	+	
			*Alternaria alternata*	+	
			*Curvularia lunata*	+	
	Petroleum ether		*Staphylococcus aureus*	−	
			*Bacillus subtilis*	−	
			*Bacillus thuringiensis*	−	
			*Enterococcus faecalis*	−	
			*Serratia marcescens*	−	
			*Pseudomonas aeruginosa*	−	
			*Proteus vulgaris*	−	
			*Proteus mirabilis*	−	
			*Klebsiella pneumoniae*	−	
			*Escherichia coli*	−	
Soxhlet	Ethanol	Disc diffusion	ESBL strains		[90]
			*Escherichia coli*	+	
			*Klebsiella pneumoniae*	−	
			Carbapenem-resistant strains		
			*Acinetobacter baumannii*	−	
			*Klebsiella pneumoniae*	+	
			*Pseudomonas aeruginosa*	+	
	Petroleum ether		ESBL strains		
			*Escherichia coli*	−	
			*Klebsiella pneumoniae*	+	
			Carbapenem-resistant strains		
			*Acinetobacter baumannii*	−	
			*Klebsiella pneumoniae*	−	
			*Pseudomonas aeruginosa*	−	
UAE	Methanol, acetone, chloroform, water	Agar well diffusion	*Bacillus cereus*	+	[73]
			*Escherichia coli*	+	
			*Staphylococcus aureus*	+	
			*Pseudomonas aeruginosa*	+	
UAE	Methanol	Disc diffusion	Microbes in fish surimi	+	[97]
MAE	Ethanol	Disc diffusion	*Streptococcus mutans*	+	[98]
			*Streptococcus mitis*	+	
			*Streptococcus pyogenes*	+	

## Data Availability

Data sharing not applicable.
